# A Web-Based Computerized Adaptive Testing (CAT) to Assess Patient Perception in Hospitalization

**DOI:** 10.2196/jmir.1785

**Published:** 2011-08-15

**Authors:** Tsair-Wei Chien, Wen-Chung Wang, Sheng-Yun Huang, Wen-Pin Lai, Julie Chi Chow

**Affiliations:** ^1^Department of ManagementChi Mei Medical CenterYungkangTaiwan; ^2^Assessment Research CenterThe Hong Kong Institute of EducationHong KongChina; ^3^Department of EmergencyChi Mei Medical CenterYungkangTaiwan; ^4^Department of PaediatricsChi Mei Medical CenterYungkangTaiwan

**Keywords:** Computerized adaptive testing, computer on wheels, classic test theory, IRT, item response theory, nonadaptive testing

## Abstract

**Background:**

Many hospitals have adopted mobile nursing carts that can be easily rolled up to a patient’s bedside to access charts and help nurses perform their rounds. However, few papers have reported data regarding the use of wireless computers on wheels (COW) at patients’ bedsides to collect questionnaire-based information of their perception of hospitalization on discharge from the hospital.

**Objective:**

The purpose of this study was to evaluate the relative efficiency of computerized adaptive testing (CAT) and the precision of CAT-based measures of perceptions of hospitalized patients, as compared with those of nonadaptive testing (NAT). An Excel module of our CAT multicategory assessment is provided as an example.

**Method:**

A total of 200 patients who were discharged from the hospital responded to the CAT-based 18-item inpatient perception questionnaire on COW. The numbers of question administrated were recorded and the responses were calibrated using the Rasch model. They were compared with those from NAT to show the advantage of CAT over NAT.

**Results:**

Patient measures derived from CAT and NAT were highly correlated (*r* = 0.98) and their measurement precisions were not statistically different (*P* = .14). CAT required fewer questions than NAT (an efficiency gain of 42%), suggesting a reduced burden for patients. There were no significant differences between groups in terms of gender and other demographic characteristics.

**Conclusions:**

CAT-based administration of surveys of patient perception substantially reduced patient burden without compromising the precision of measuring patients’ perceptions of hospitalization. The Excel module of animation-CAT on the wireless COW that we developed is recommended for use in hospitals.

## Introduction

As computer technology and health care become more integrated, many hospitals have adopted mobile nursing carts that can be easily rolled up to a patient’s bedside to access charts and help nurses perform their rounds [1-3]. Besides increasing efficiency by including basic functions such as billing records and decreasing the number of trips nurses need to take to the medication room [3], the carts can reduce patient burden by allowing them to answer questions on activities of daily living using computerized adaptive testing (CAT) [1]. However, few papers have reported data regarding the bedside use of wireless computers on wheels (COW) to collect questionnaire-based information on their perception of hospitalization on discharge from the hospital. Collecting patients’ feedback on their perspectives has become an important part of patient involvement and participation for health caregivers; thus, this question is important [4-6].

### Gathering Feedback Efficiently From Patients

Two new modes of survey administration have been reported to make surveys more easily accessible to those who cannot read or write [7]. These include using automated telephone technology through an interactive voice response system and using Internet-like visualizations to complete questionnaires online. In medical practice, hospital staff usually hand a questionnaire to patients at the end of their visit and ask them to complete it prior to leaving hospital. At the Picker Institute Europe [5], questionnaires are sent annually to a randomized list of eligible patients who had been discharged from the hospital. Both of these methods are less prompt and efficient than using wireless COW to collect data on patients’ perspectives on being discharged from the hospital.

### Computer Assessment and Computer-Adaptive Testing

There is no doubt that using wireless COW at a patient’s bedside is an efficient way of instantly gathering feedback from patients. Traditional paper-and-pencil or computer-based devices (nonadaptive testing [NAT]) impose a large respondent burden because patients are required to answer all the questions. In contrast, CAT-based tests developed using item response theory (IRT) [8] can achieve a similar degree of measurement precision to NAT using only about half the test length [1,9-11]. Most studies investigating IRT- and CAT-based tests have evaluated both efficiency and precision for CAT-based tests with dichotomous items. Whether CAT-based tests with polytomously scored items (CAT as defined in this study) can be incorporated with wireless COW in hospitals for gathering feedback from patients should be investigated.

### Rasch Analysis

In classical test theory, raw scores (or linear transformation scores, eg, T score) are usually adopted as respondent measures. However, subsequent parametric statistical analyses, such as computing mean, variance, correlation coefficient, and Cronbach alpha [12,13], would be incorrect because raw scores are not on an additive interval scale [14].

To overcome this obstacle, the IRT-based Rasch model [15], a probabilistic relationship between a person’s level of a latent trait (commonly referred to as ability or measure) and an item’s property (difficulty or threshold), was developed. Both person ability and item difficulty (calibrated in terms of log odds or logits) are located along the same continuum. A useful scale (or questionnaire) is usually examined by 3 important criteria for the Rasch model, namely, unidimensionality, item fit, and item invariance (or so-called differential item functioning [16]). These criteria are detailed in Smith et al [17]. There are many published papers [1,18-21] of studies using the Rasch model to develop CAT in clinical settings, but none of them have incorporated the Internet-based polytomously scored CAT to gather feedback from patients in hospitals.

### Objectives

The purpose of this study was to evaluate the relative efficiency of an Internet-based polytomously scored CAT and the precision of CAT-based measures of perceptions of hospitalized patients, as compared with those measured by NAT. An Excel (Microsoft Corporation, Redmond, WA, USA) module of our CAT multicategory assessment is provided as an example.

## Methods

### Data collection

#### Participants

The study sample was recruited from inpatients at a 1333-bed medical center in southern Taiwan. Patients who had been discharged were selected randomly by the digit code of their invoice number during each morning and afternoon interval from Monday through Friday in summer 2010.

#### Procedure

As an incentive for participation, patients were offered a gift card for US $3.20 good for purchases at 7-11 convenience stores. A total of 200 patients either completed the questionnaire on COW themselves or were helped by a trained volunteer (if they were unable to personally complete the questionnaire); proxies were allowed because most of those helping patients carry out their discharge procedure were the patients’ family members or friends. Time spent by each patient was recorded in Excel after they completed the questionnaire. This study was approved and monitored by the Research and Ethical Review Board of the Chi-Mei Medical Center, Tainan, Taiwan.

#### Tool: CAT-Format Questionnaire

We designed the 18-item CAT questionnaire in Excel based on an 18-item inpatient perception questionnaire (IPQ-18) [5]; see Table 1). Unidimensionality, local independence, item fit, and differential item functioning using the Rasch model to investigate these criteria have been previously reported [5].

Data collected from the patients included demographic characteristics (gender, treatment department, age, and person completing survey, ie, proxy or patient); perception measure in a logit unit; number of items needed to be completed; and mean square errors (MNSQ) of infit and outfit (indicators of response patterns for the IPQ-18 scale [5]) (see Table 1, Multimedia Appendix 1, and Multimedia Appendix 2).

**Table 1:** Items of the 18-item scale ordered by item overall difficulties

Item number	Scale content		Difficulty				
	Category^a^	Item	Overall	Step1	Step2	Step3	Step4
39	L	Did staff tell you about medication side effects when going home?	3.78	0.02	1.87	5.35	7.89
41	L	Did doctors or nurses give your family information needed to help you?	2.76	–1.00	0.85	4.33	6.87
27	N	Did hospital staff talk about your worries and fears?	2.22	–1.54	0.31	3.79	6.33
11	W	Were you ever bothered by noise at night from other patients?	1.58	–2.18	–0.33	3.15	5.69
24	N	Were you involved in decisions about your care and treatment?	0.67	–3.09	–1.24	2.24	4.78
30	N	How long was it after using the call button before you got the help you needed?	0.42	–3.34	–1.49	1.99	4.53
42	L	Did staff tell you how to contact them if worries arose after leaving?	–0.3	–4.06	–2.21	1.27	3.81
9	A	Did you feel you waited a long time to get to a bed on a ward?	–0.63	–4.39	–2.54	0.94	3.48
44	O	How would you rate how well the doctors and nurses worked together?	–0.71	–4.47	–2.62	0.86	3.4
2	A	How organized was the care you received in the emergency room?	–0.95	–4.71	–2.86	0.62	3.16
5	A	Were you given enough notice of your date of admission?	–1.08	–4.84	–2.99	0.49	3.03
12	W	Were you bothered by noise at night from hospital staff?	–1.1	–4.86	–3.01	0.47	3.01
17	D	Did you have confidence and trust in the doctors treating you?	–1.1	–4.86	–3.01	0.47	3.01
23	N	Did staff say one thing and something quite different happened to you?	–1.1	–4.86	–3.01	0.47	3.01
38	L	Did staff explain the purpose of the medicines so that you could understand?	–1.1	–4.86	–3.01	0.47	3.01
18	D	Did doctors talk in front of you as if you weren’t there?	–1.12	–4.88	–3.03	0.45	2.99
19	N	Did you get answers that you could understand from a nurse?	–1.12	–4.88	–3.03	0.45	2.99
34	P	Did hospital staff do everything they could to help you control your pain?	–1.12	–4.88	–3.03	0.45	2.99

^a^ Categories are A: admission to hospital; D: doctors; L: leaving hospital; N: nurses; O: overall; P: pain; W: hospital and ward.

### CAT Procedure

#### Outfit and Infit Statistics

Outfit statistics are based on unweighted sum of squared standardized residuals and are sensitive to unexpected outlying, off-target, and low-information responses; whereas infit statistics are based on weighted sum of squared standardized residuals and are sensitive to unexpected inlying patterns among informative and on-target observations [22]. Smith [23] found that Rasch outfit MNSQ approaching 1.0 [24] demonstrates a higher power than its counterpart of infit MNSQ. Outfit MNSQ of 2.0 or greater for a patient indicate a possibly aberrant response pattern [24].

#### CAT Procedures and Features

**Figure 1:**
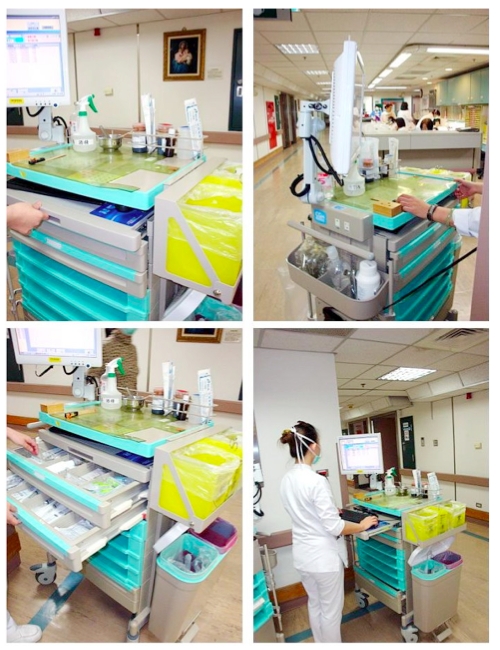
Using a wireless computer on wheels (COW) to collect data on patients’ perspectives on hospitalization

**Figure 2:**
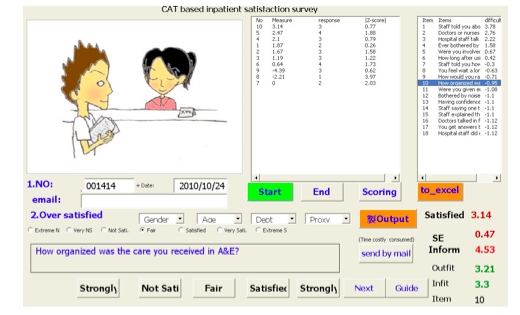
Snapshot of computerized adaptive testing (CAT)-based inpatient perception questionnaire for patients

We programmed a Visual Basic for Applications (VBA) module in Microsoft Excel and on the Internet (http://www.healthup.org.tw/cat.asp, http://www.webcitation.org/60xWv6p6d) complying with several rules and criteria for CAT-based testing on COW (Figure 1, Figure 2). The person separation reliability (similar to Cronbach alpha) calculated from the original paper [5] was 0.94 (mean 2.64, SD 2.09). Based on this number, the CAT stop rule for measurement of standardized error was determined to be 0.51(SD × sqrt(1 – alpha) = 2.09 × sqrt(1 – 0.94)).

We also set another stop rule so that the minimum number of questions required for completion was 10 items (10/18, 56%), because CAT achieves a similar measurement precision to NAT with only about half the test length [1,9-11]. The initial question was selected from the pool of 18 items according the patient’s overall perception of satisfaction in their hospitalization. The provisional person measure is estimated by the maximum of the log-likelihood function using an iterative Newton-Raphson procedure [1,10] (Multimedia Appendix 2) after 3 items were responded to, without all answers corresponding to either 0 or 4. The next question selected was the one with the highest information value among the remaining unanswered questions weighted against the provisional person measure. The details of CAT procedures are shown in Multimedia Appendix 2 and Multimedia Appendix 3.

### Comparison of Efficiency Between NAT and CAT

Two indicators used to examine CAT efficiency in this study are (1) whether the number of questions needed was significantly less than for NAT (18 questions) and (2) whether the precision of person measures was less than that for NAT. We used paired *t* tests to make these two statistical inferences.

Accordingly, the perception measure based on NAT should be estimated in advance for each patient who was assumed to have answered all 18 items. The following steps were thus followed: (1) we used a standard item response-generation method [25-29] to generate responses to 18 questions for each patient with given question difficulty parameters (in Table 1) and a patient perception measure (by CAT), and (2) we applied the rectangle metric of 18 questions × 200 persons to re-estimate NAT measures for each patient using WINSTEPS software (WINSTEPS version 3.72.0: Winsteps.com, Chicago, IL, USA) (the 18-question difficulties are anchored in WINSTEPS with iafile shown in Multimedia Appendix 2).

### Statistical Analysis

SPSS software for Windows (Version 12, SPSS, Chicago, IL) was used for all statistical analysis.

#### Descriptive Statistics

Data on patient gender, age, treatment department, and proxy respondent were collected. Noncontinuous variables were reported as frequency and percentages, and were examined by chi-square tests.

#### Analytic statistics

For continuous variables, CAT and NAT measures were compared using the Pearson correlation coefficient. Patient perception measures obtained by CAT were compared between groups using *t* tests or analysis of variance (ANOVA). Time spent by patients was averaged by using logarithmic transformation and reported as mean (SD) by exponential function. All analyses were considered to be statistically significant at the .05 alpha level.

## Results

As seen in Table 2, there were no significant associations between gender and other demographic characteristics (ie, treatment department, age, and participant). Among inpatients we approached, 13% (31/231) were unwilling to respond to the CAT questions due to personal reasons, despite the incentive we offered. CAT and NAT measures were highly correlated (*r* = 0.98).

**Table 2:** Demographic characteristics of the study population (N = 200)

Variable		Male		Female		Total	χ^2^_(r-1)*(c-1)_	
		n	%	n	%		Test	*P* value
**Respondent**							0.6	.45
	Willing to participate	100	50	100	50	200		
	Unwilling to participate	13	42	18	58	31		
**Age(years)**							0.9	.82
	≤16	31	31	25	25	56		
	17–40	27	27	30	30	57		
	41–70	25	25	27	27	52		
	>70	17	17	18	18	35		
**Department**							3.9	.42
	Internal medicine	44	44	41	41	85		
	Surgery	28	28	22	22	50		
	Obstetrics and gynecology	8	8	14	14	22		
	Pediatrics	11	11	7	7	18		
	Other	12	12	16	16	28		
**Participant/proxy**							1.1	.57
	Family	75	75	81	81	156		
	Friend	15	15	12	12	27		
	Patient	10	10	7	7	17		

Mean time spent by patients in CAT was 54.91 seconds (SD 8.00; maximum 76; minimum 33). As shown in Table 3, CAT required substantially fewer questions than NAT (*P* < .001). NAT required all of the 200 patients to respond to all 18 questions, and thus yielded a total of 3600 responses. In CAT, a total of 2083 responses were required, meaning that on average a patient answered 10.42 questions. Thus, as compared with NAT, CAT received an efficiency gain in test length of 0.42 (defined as 1 – ratio of total responses by CAT and NAT: 1 – 2084/3600).

**Table 3:** Comparison of computerized adaptive testing (CAT) versus nonadaptive testing (NAT) (all questions having to be answered) in efficiencya as assessed by paired t test

		Mean	Variance	Response	Maximum	Minimum	Paired *t*_199_ test	*P* value
**Test length(number of questions answered)**								
	NAT	18	0.00	3600^b^	18	18	–476.72	<.001
	CAT	10.42	0.25	2084^b^	12	10		
**Estimatedmeasures(mean and variance)**								
	NAT	0.69	2.66	3600	4.16	–2.69	1.10	.14
	CAT	0.71	2.62	2084	4.00	–2.56		
**Timespent (seconds)**								
	CAT	54.91^c^	64.04^c^	2084	76^3^	33^3^		

^a^Efficiency = (1 – 2084/3600) = 0.58.

^b^3600 = 200 × 18; 2084 = 200 × 10.42.

^c^On second unit.

Regarding precision of measurement, person measures from CAT did not statistically differ from those from NAT (*P* = .14). ANOVA revealed that patient perception measures did not differ between groups across elements; *t* test analyses showed that they also did not differ between genders (Table 4).

**Table 4:** Comparison of inpatient perception by demographic characteristic

Variable		Male		Female		ANOVA^a^	
		Mean	SD	Mean	SD	Test	*P* value
Proportion		0.77	1.59	0.65	1.66	*t*_398_ = 0.55	.59
**Age(years)**						*F*_3,196_ = 0.71	.55
	≤16	0.77	1.72	0.83	1.81	–0.12	.89
	17–40	1.23	1.54	0.58	1.40	1.68	.09
	41–70	0.72	1.48	0.53	1.74	0.42	.67
	>70	0.13	1.45	0.69	1.83	–1.00	.32
**Department**						*F*_4,195_ = 0.92	.45
	Internal medicine	0.65	1.53	0.49	1.48	0.47	.63
	Surgery	0.61	1.56	0.9	1.77	–0.77	.44
	Obstetrics and gynecology	1.00	1.91	0.77	1.70	0.28	.77
	Pediatrics	0.45	1.79	0.19	2.00	0.30	.78
	Other	1.73	1.29	0.68	1.85	1.67	.11
**Participant/proxy**						*F*_2,197_ = 0.36	.69
	Family	0.90	1.58	0.60	1.62	1.14	.25
	Friend	0.58	1.60	0.93	2.10	–0.49	.62
	Patient	0.16	1.62	0.72	1.43	–0.73	.47

^a^ Analysis of variance.

Total person mean 0.71 logits (SD 1.62); median 0.59; coefficient of skewness 0.103 (*P* = .54); coefficient of kurtosis –0.89 (*P* = .03); D’Agostino-Pearson test for normal distribution accept normality (*P* = .09).

## Discussion

### Key Finding

The results from this study indicate that CAT-based testing using COW can reduce patient (or proxy) burdens. It is up to 42% more efficient in answering questions and achieves a very similar degree of measurement precision to NAT.

### What This Adds to What Was Known

Consistent with the literature [1,9-11,30], the efficiency of CAT was supported. We confirmed that the CAT-based IPQ-18 on COW requires significantly fewer questions to measure patient perception than NAT, but does not compromise precision of measurement.

### What is the Implication, What Should be Changed

Using an Excel module of animation for CAT on COW as a tool that can help hospital staff efficiently and precisely gather feedback from patients is technically feasible. Outfit MNSQ of 2.0 or greater can be used to examine whether patient responses are distorted or abnormal—that is, many more responses unexpectedly did not fit the model’s requirement and were deemed to be very likely to be careless, mistaken, or awkward [1,5,6,24]. Thus, CAT makes detection of problematic responses easier—normally, inspecting problematic feedback from patients using classical test theory is rather difficult.

### Strength of This Study

There are 2 major forms of standardized assessments in clinical settings [31]: (1) a lengthy questionnaire to achieve a precise assessment that requires significant amounts of time and training to administer, and (2) a rapid short-form scale that briefly screens for the most common symptoms using cut-off points to identify degrees of impairment [32,33]. CAT has the advantages of both forms: precision and efficiency. This paper reports several achievements, including using the Rasch rating scale model [34] (instead of dichotomy) to design CAT in a perception survey, applying CAT on a COW, and offering an Excel module with an animation prototype (demonstrated in Multimedia Appendix 2 or http://www.healthup.org.tw/cat.asp). This module and available files can complement the limited uses for interactive voice response or Internet-like visualization online [7] to complete questionnaires and put them into clinical practice.

We conducted an actual CAT-based survey instead of CAT with simulations. This study demonstrates the whole procedure of a CAT-based survey, from the beginning of data collection (Figure 2and Multimedia Appendix 3) through the end of the evaluation report (Table 4), and fulfills the goal of creating a Web-CAT with graphs and animations, as stated in our previous paper [35].

### Limitations of the Study

Several issues should be considered more thoroughly in further studies. First, a total of 200 patients were surveyed on the IPQ-18. The generalizability of this study needs to be investigated with more studies on different samples and different instruments. Second, there is a potential sampling bias in this study. Those who completed the IPQ-18 CAT on COW tended to be younger; and proxies were used to represent patients to complete the discharge procedure from hospital, because they were selected randomly by the digit code of their invoice number on the patient’s discharge. The proportion of proxies, who are assumed to be healthier and more capable of filling out a questionnaire, was very high (183/200, 91.5%; see Table 2). This sample therefore does not reflect mostly the patients’ perspective on hospitalization, which possibly affects the study results shown in Table 4. Third, the patient burden was determined by the number of questions administered in this study. Other indices may be collected where available, such as time and effort required for test administration, and accessibility of the hospital [33, 34].

In addition, we set at least 10 items in CAT to be completed as one of the stop rules, which might inflate the test length to some extent. As a result, the test length of CAT was about 58% that of NAT, a little higher than in previous studies with about half the test length [1,9-11].

### Applications

A large variety of behavior-change techniques and other methods to promote exposure to interventions have been used [36]. There are concerns about how to entice patients (or proxies) to complete surveys before they are discharged from the hospital. Offering reward points or coupons good for credits toward another service is recommended because perception surveys are not similar to other clinical scales conducted by clinicians, where patients themselves consider the benefits to their health.

A telephone survey with CAT-based administration or patient self-report on the Internet (demonstrated at http://www.healthup.org.tw/cat.asp) can be combined with the CAT on COW for gathering feedback from patients easily, quickly, and efficiently.

There are many issues that should be addressed in the future, including studies that address the limitations noted above. For example, using CAT on COW at patients’ bedsides to gather their feedback before discharge from the hospital can solve the problem of sampling bias (eg, when proxies constitute a high proportion of respondents) and warrants further study. Surveying perceptions of hospital service via the Internet by CAT-type telephone or self-report is encouraged to complement CAT on COW and questionnaires delivered by mail to discharged patients, such as the Picker Institute Europe’s annual survey.

One of the important advantages of CAT scoring is that the item pool can be expanded without changing the metric [37]. CAT administrators may expand the IPQ-18 item pool or replace items with other kinds of questions as presented in the Excel spreadsheet example. It must be noted that (1) overall item and step (threshold) difficulties of the questionnaire must be calibrated in advance using Rasch analysis (eg, the IPQ-18 of this study was examined by Rasch analysis in a previous paper [5]), and (2) picture and voice files for each question should be well prepared in an appropriate folder that can be shown simultaneously with the corresponding question in an animation module of CAT.

### Conclusion

CAT-based administration of surveys of patient perception reduces patient burden without compromising measurement precision. The Excel module for animation-CAT on COW connected to a mainframe computer is recommended for assessing patients’ perceptions of their experience in the hospital.

## Multimedia Appendix 1

Excel VBA module for CAT delivering results to the website through an Internet address

## Multimedia Appendix 2

Comprehensive overview of Rasch models and the CAT process

## Multimedia Appendix 3

Screenshot of the module with an animation-CAT design

## Abbreviations

ANOVA: analysis of variance
CAT: computerized adaptive testing
COW: computers on wheels
IPQ: inpatient perception questionnaire
IRT: item response theory
MNSQ: mean square errors
NAT: nonadaptive testing
VBA: Visual Basic for Applications
