# Supplementary figures and images for: A Web-Based Computerized Adaptive Testing (CAT) to Assess Patient Perception in Hospitalization
Source: J Med Internet Res. 2011 Aug 15;13(3):e61. doi: 10.2196/jmir.1785 (PMC3222179; doi:10.2196/jmir.1785)

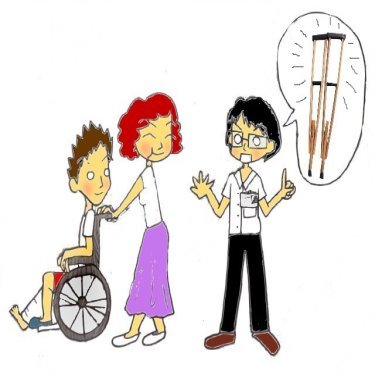

Supplement: Supplementary file 1 [file jmir_v13i3e61_app1.zip › cat/2.jpg]

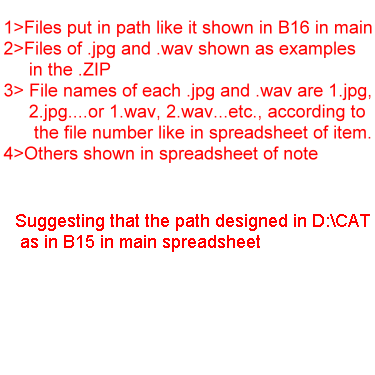

Supplement: Supplementary file 1 [file jmir_v13i3e61_app1.zip › cat/nopic.gif]

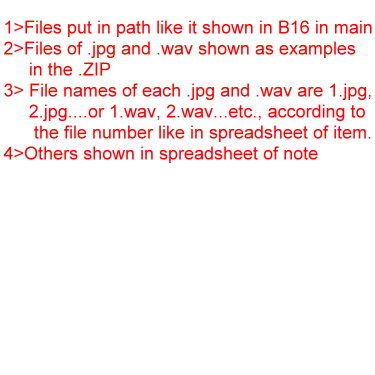

Supplement: Supplementary file 1 [file jmir_v13i3e61_app1.zip › cat/nopic.jpg]
