# Supplementary material for: A Web-Based Computerized Adaptive Testing (CAT) to Assess Patient Perception in Hospitalization
Source: J Med Internet Res. 2011 Aug 15;13(3):e61. doi: 10.2196/jmir.1785 (PMC3222179; doi:10.2196/jmir.1785)
Supplement: Supplementary file 2 [file jmir_v13i3e61_app2.pdf]

A Web-Based Computerized Adaptive Testing (CAT) to Assess Patient Perception in Hospitalization

Tsair-Wei Chien, Wen-Chung Wang

Features

- CAT-format questionnaire come from a previous paper
- Step threshold difficulties yielded
- Item bank prepared
- Logic of CAT
- The Rasch rating scale model
- A Web-Based Computerized Adaptive Testing (CAT)
- Newton-Raphson method, information, SE and outfit
- Select & present optimal scale item
- A screencast of the module
- CAT item-by-item selection report for an examinee
- How to simulate Rasch data
- Control file of WINSTEPS to gain measures of NAT
- Comparison of CAT and NAT in efficiency

CAT-format questionnaire

- An IPQ-18 presented in a previous paper:

  Chien TW, Wang WC, Wang HY, Lin HJ: Online assessment of patients' views on hospital performances using Rasch model's KIDMAP diagram. ***BMC Health Serv Res. 2009***;9:135. http://www.biomedcentral.com/1472-6963/9/135

Step (threshold) difficulties are yielded

- The category Rasch-Andrich thresholds (step difficulties) were ordered as **-3.76, -1.91, 1.57 and 4.11**. http://www.biomedcentral.com/1472-6963/9/135
- Ex. Overall difficulty=3.78 for item 39 yields step difficulty as followings:

step 1= 0.02[3.78+(-3.76)],

step 2=1.87[3.78+(-1.91)],

step 3=5.35[3.78+1.57],

step 4=7.89[3.78+4.11].

Item bank prepared in Excel

Item_no	Items	Overall difficulty	Step difficulties b1	b2	b3	b4	desp1	desp2	desp3	desp4	desp5
1	Staff….	3.78	0.02	1.87	5.35	7.89	Strongly NS	Not Satisfied	Fair	Satisfied	Strongly S
2	Docto….	2.76	-1	0.85	4.33	6.87	Strongly NS	Not Satisfied	Fair	Satisfied	Strongly S
3	Hospi….	2.22	-1.54	0.31	3.79	6.33	Strongly NA	Not Agree	Fair	Agree	Strongly A
4	Ever ….	1.58	-2.18	-0.33	3.15	5.69	Hard to	Tolerance	Fair	A little	None
5	Were ….	0.67	-3.09	-1.24	2.24	4.78	Strongly NA	Not Agree	Fair	Agree	Strongly A
6	How l….	0.42	-3.34	-1.49	1.99	4.53	Strongly NS	Not Satisfied	Fair	Satisfied	Strongly S
7	Staff….	-0.3	-4.06	-2.21	1.27	3.81	Strongly NS	Not Satisfied	Fair	Satisfied	Strongly S
8	You f….	-0.63	-4.39	-2.54	0.94	3.48	Strongly NS	Not Satisfied	Fair	Satisfied	Strongly S
9	How w….	-0.71	-4.47	-2.62	0.86	3.4	Strongly NS	Not Satisfied	Fair	Satisfied	Strongly S
10	How o….	-0.95	-4.71	-2.86	0.62	3.16	Strongly NS	Not Satisfied	Fair	Satisfied	Strongly S
11	Were ….	-1.08	-4.84	-2.99	0.49	3.03	Strongly NS	Not Satisfied	Fair	Satisfied	Strongly S
12	Bothe….	-1.1	-4.86	-3.01	0.47	3.01	Hard to	Tolerance	Fair	A little	None
13	Havin….	-1.1	-4.86	-3.01	0.47	3.01	None	Hard to	Fair	A little	Trust
14	Staff….	-1.1	-4.86	-3.01	0.47	3.01	Strongly NA	Not Agree	Fair	Agree	Strongly A
15	Staff….	-1.1	-4.86	-3.01	0.47	3.01	Strongly NA	Not Agree	Fair	Agree	Strongly A
16	Docto….	-1.12	-4.88	-3.03	0.45	2.99	Strongly NA	Not Agree	Fair	Agree	Strongly A
17	You g….	-1.12	-4.88	-3.03	0.45	2.99	Strongly NA	Not Agree	Fair	Agree	Strongly A
18	Hospi….	-1.12	-4.88	-3.03	0.45	2.99	Strongly NA	Not Agree	Fair	Agree	Strongly A

Logic of CAT

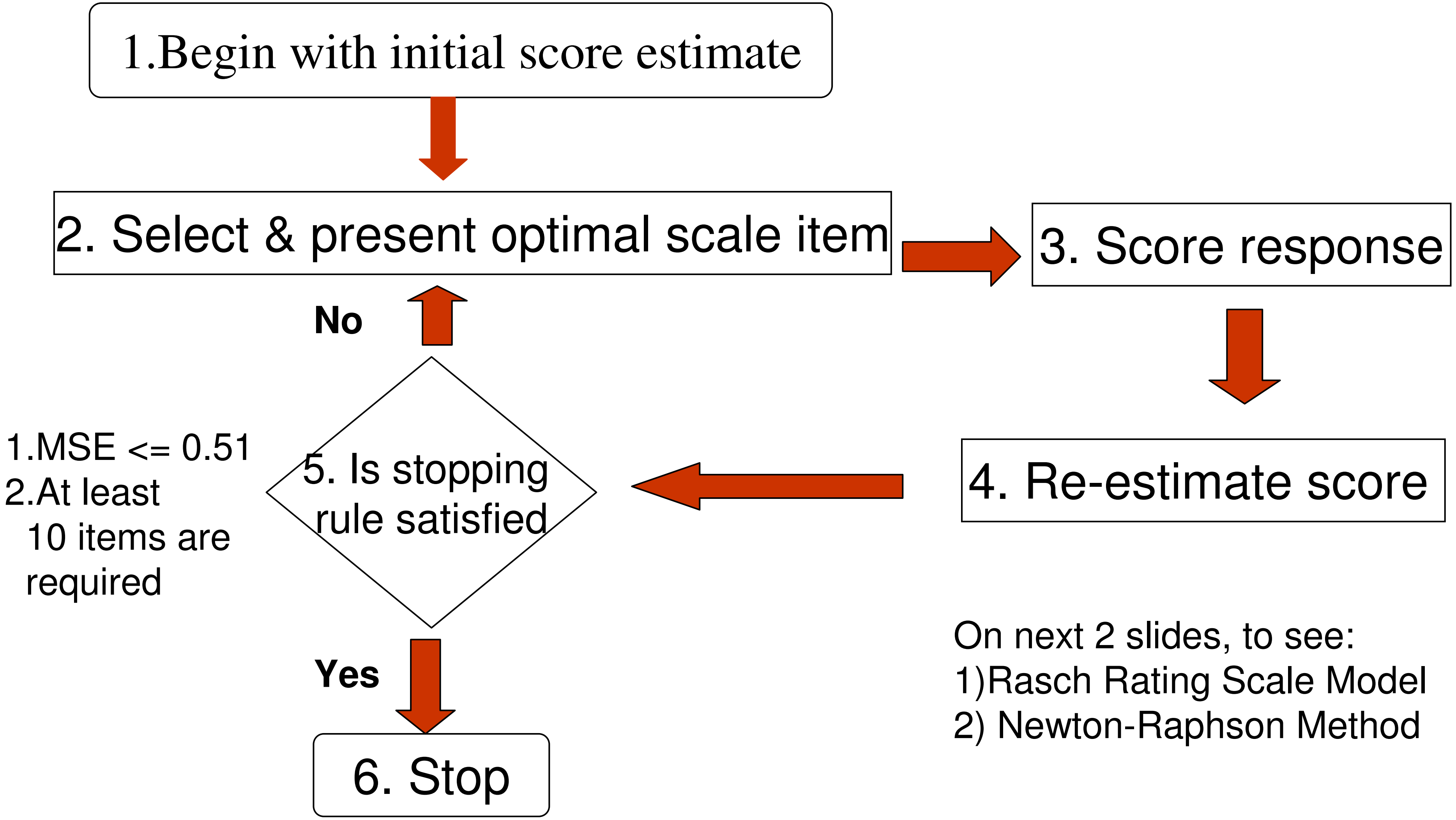

On next 2 slides, to see:
1)Rasch Rating Scale Model
2) Newton-Raphson Method

The Rasch rating scale model (RSM)

Overall difficulty

Step difficulty

Person ability

$$P(X_{ni} = x) = \frac{\exp \sum_{j=0}^{x} [\beta_n - (\delta_i + \tau_j)]}{\sum_{k=0}^{m} \exp \sum_{j=0}^{k} [\beta_n - (\delta_i + \tau_j)]}, x = 0,1,\dots m$$

Where P(Xni = x) is the probability that a person n is assigned to rating scale category x on item i, each item has m + 1 rating scale categories

And

$$\sum_{j=0}^{0} \left[\beta_n - (\delta_i + \tau_j)\right] = 0$$

Newton-Raphson method, information, SE and outfit

- **To re-estimate score** (When provisional estimate of a person and item difficulties known)
- for Newton=1 to n
- for item=1 to selected item
- first_order = first_D + (obs. score- expected score)------------------------------------→
- second_order = second_D + variance
- information = information + variance --→
- next item
- **SE** = 1 / information^ 0.5
- Ability = Ability - first_order / second_order
- Convergency to a limit
- if abs(first_order / second_order)<.05 then exit for
- next Newton
- ==

Stop rule

- **To obtain the probability,variance and outfit MNSQ**
- a) Compute the cumulative exponential of observing each category, ex. A1,a1… was shown in previous slide
- b) ALL_p = a0 + a1 + a2 + a3 + a4
- c)The expected score for each item = (0) * a0 / all_p + (1) * a1 / all_p + (2) * a2 / all_p + (3) * a3 / all_p + (4) * a4 / all_p
- d)The variance for each item is stated as:
- variance = a0 / all_p * (0 - ex) ^ 2 + a1 / all_p * (1 - ex) ^ 2 + a2 / all_p * (2 - ex) ^ 2 + a3 / all_p * (3 - ex) ^ 2 + a4 / all_p * (4 - ex) ^ 2
- Zsquare = (obs. score- ex) ^ 2 /var
- for j=1 to selected item
- outfit = outfit + Zsquare(i)
- next j

Select & present optimal scale item by Select the maximum information for unselected items

Information=variance = a0 / all_p * (0 - ex) ^ 2 + a1 / all_p * (1 - ex) ^ 2 + a2 / all_p * (2 - ex) ^ 2 + a3 / all_p * (3 - ex) ^ 2 + a4 / all_p * (4 - ex) ^ 2

- expected score for each item = (0) * a0 / all_p + (1) * a1 / all_p + (2) * a2 / all_p + (3) * a3 / all_p + (4) * a4 / all_p

$$P(X_{ni} = x) = \frac{\exp\sum_{j=0}^{x}[\beta_n - (\delta_i + \tau_j)]}{\sum_{k=0}^{m}\exp\sum_{j=0}^{k}[\beta_n - (\delta_i + \tau_j)]}, x = 0,1,...m$$

A screencast of the module in Excel

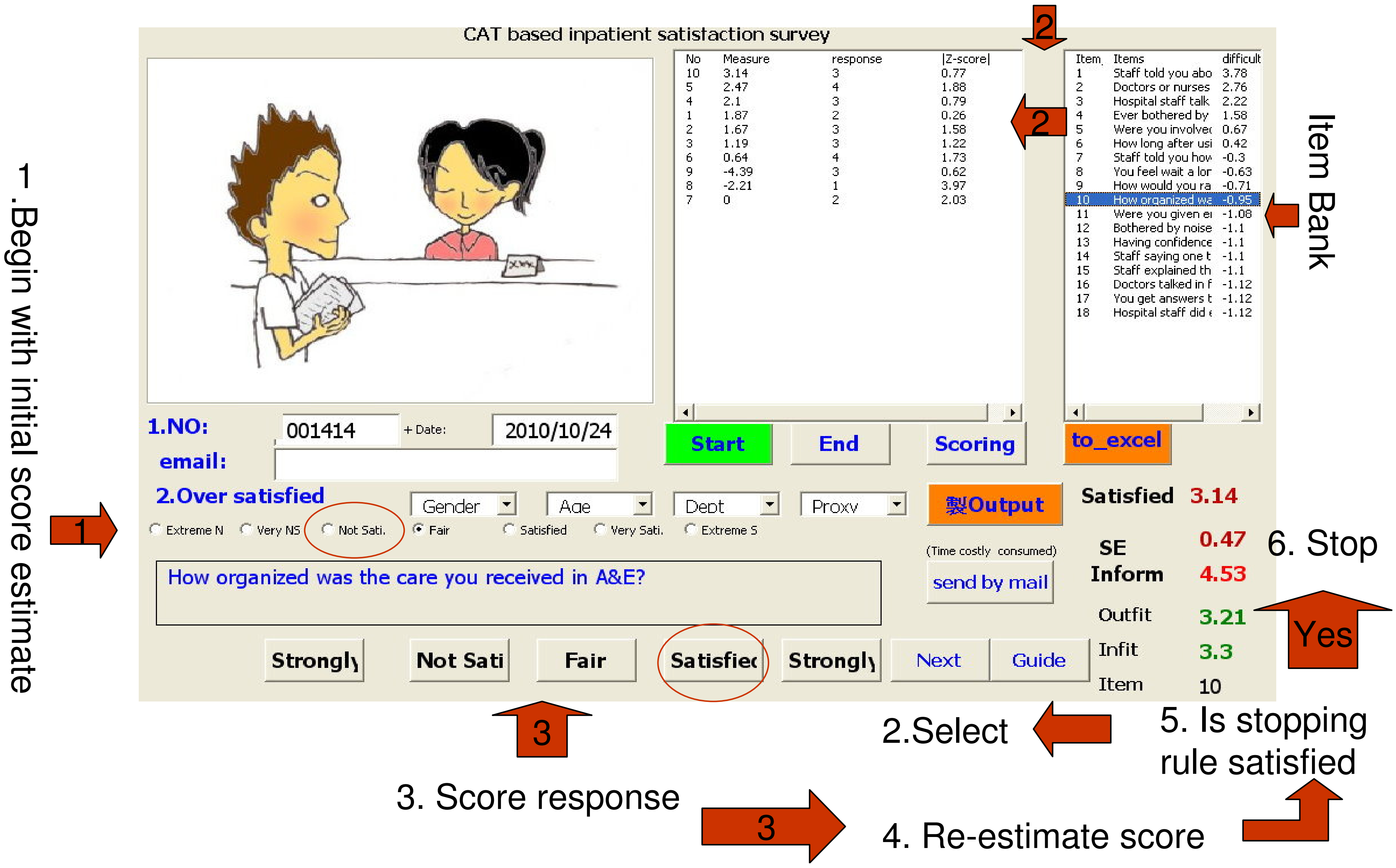

A web-based computerized adaptive testing (CAT)

- http://www.healthup.org.tw/cat.asp

Inpatient survey on CAT

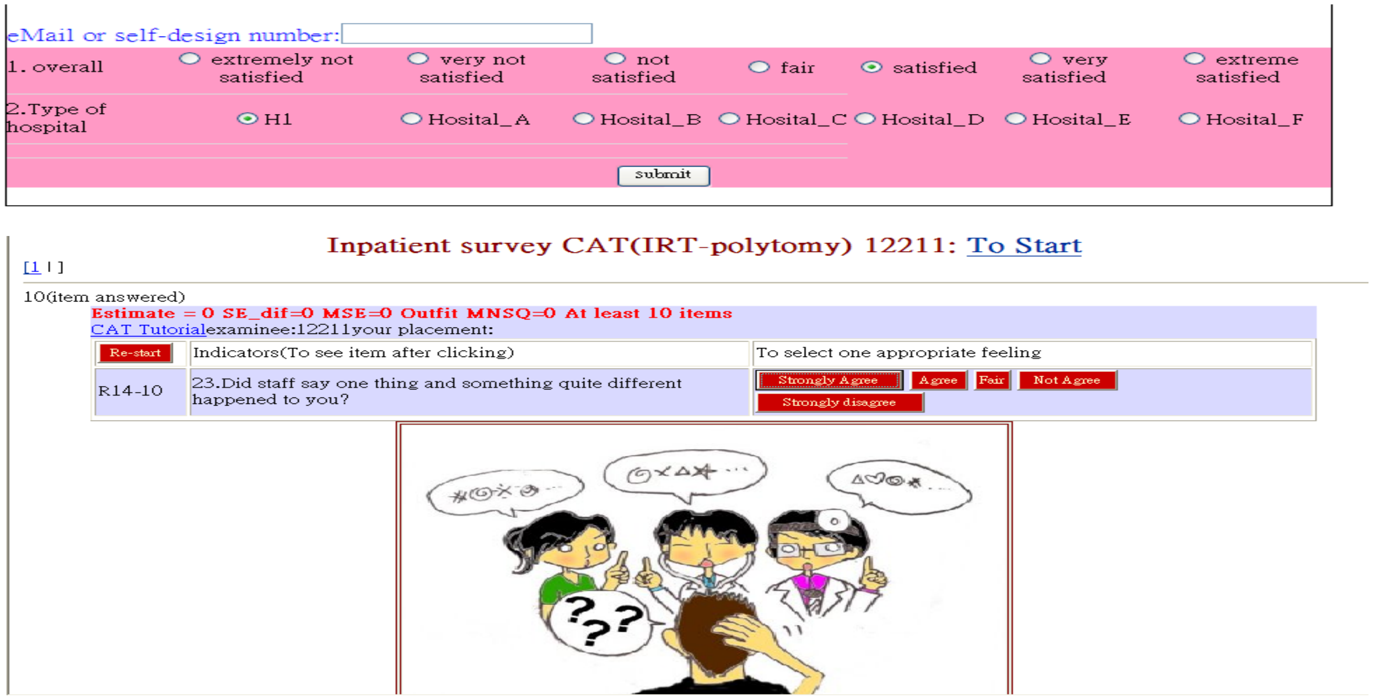

CAT item-by-item selection report for an examinee

This test terminated when the SE less than 0.5

Minimum number of items = 10 | Maximum number of items = 18

Examinee Name: | Theta was estimated by maximum likelihood

Examinee ID: 001412 | The standard error band plotted as that pluses mor minumes MSE around theta

Date tested: ######## | X=initial theta value C=Correct answer I=incorrect answer

Item	Theta	SE	-6 -5 -4 -3 -2 -1 0 1 2 3 4 5 6	|Z|
7	0	0	----------3--	>
6	1.27	0	----------2--	>
8	-1.49	0	----------1--	2.41*
9	0.2	0.67	-------3------	
4	0.36	0.62	------2-----	
5	0.72	0.59	------3-----	
3	0.85	0.55	------2-----	
1	0.86	0.52	-----1----	
2	0.52	0.49	-----0----	
10	0.53	0.47	-----2----	

>Arbitrarily assigned value, These values were not used to terminate the test.

* |Z|>2 denotes that unexpected response occurred

The final theta estimate based on the tested items was 0.53 with a standard error of 0.47,

resulting in a standard error band of 6.00000000000001E-02 to 1

How to simulate Rasch data

- **Person measures and item difficulties (including step difficulties) known after CAT**
- **How to Simulate Rasch Data**

	A	B	C	D	E	F	G	H	I	J	K	L
1	4	3.78	2.76	2.22	1.58	0.67	0.42	-0.3	-0.63	-0.71	-0.95	-1
2	3.14	1	1	2	2	2	3	2	3	3	2	
3	3.88	3	3	2	2	3	3	4	4	4	4	
4	2.17	1	1	2	3	2	3	4	3	3	3	
5	-0.03	0	1	2	1	2	2	2	2	2	2	
6	3.5	2	3	2	2	3	3	4	3	4	3	
7	4.3	3	3	2	4	3	3	4	4	4	3	

Using a item response generation method that has been introduced at **http://www.rasch.org/rmt/rmt213a.htm** to generate 18-question responses for each patient when question difficulties and patient perception measure (by CAT) are known.

WINSTEPS control file to yield measures of NAT

- &INST ; this starts the control specifications; it is optional
- TITLE = 'LIKING FOR SCIENCE (Wright & Masters p.18)'
- NI = 18 ; 25 items
- ITEM1 = 1 ; responses start in column 1 of the data
- NAME1 = 12 ; person-label starts in column 28 of the data
- ITEM = ACT ; items are called "activities"
- PERSON= KID ; persons are called "kids"
- CODES = 0123456789 ; valid response codes (ratings) are 0, 1, 2
- CLFILE=* ; label the response categories
- 0 Dislike ; names of the reponse categories
- 1 Neutral
- 2 Like
- * ; "*" means the end of a list
- IAFILE=*
- 1 3.78
- 2 2.76
- 3 2.22
- 4 1.58
- 5 0.67
- 6 0.42
- 7 -0.3
- 8 -0.63
- 9 -0.71
- 10 -0.95
- 11 -1.08
- 12 -1.1
- 13 -1.1
- 14 -1.1
- 15 -1.1
- 16 -1.12
- 17 -1.12
- 18 -1.12
- *
- NUMB=Y
- &END ; this ends the control specifications

;data shown as followings:

- 021222211223232222
- 221222133333333442
- …….

Using the 18-question response to re-estimate NAT responses for each patient using WINSTEPS software [17] (when 18-question difficulties are known using WINSTEPS anchored command file Shown In left, so that the comparison. in efficiency can be made in next slide.

Comparison of CAT and all answered items (NAT) in efficiency with paired t-test

	Mean	Variance	Response	Maximum	Minimum	t	*Prob.>Z*
Test length:							
NAT	18	0.00	3600	18	18	-476.72	< .001
CAT	10.42	0.25	2084	12	10		
Estimated ability:							
NAT	0.69	2.66	3600	4.16	-2.69	1.10	0.14
CAT	0.71	2.62	2084	4.00	-2.56		
